# Supplementary material for: Comprehensive database and evolutionary dynamics of U12-type introns
Source: Nucleic Acids Res. 2020 Jun 2;48(13):7066–78. doi: 10.1093/nar/gkaa464 (PMC7367187; doi:10.1093/nar/gkaa464)
Supplement: gkaa464_Supplemental_File [file gkaa464_supplemental_file.pdf]

Organism	Number of U12-Type Introns	Genes with Multiple U12-Type Introns	Proportion of Introns That Are U12-Type	Probability That U12-Type Introns Are Randomly Distributed
Glycine max	521	25	0.0025	2.3E-10
Arabidopsis thaliana	274	16	0.0025	3.6E-08
Zea mays	282	8	0.0017	0.0011
Oryza sativa	281	12	0.0024	7.5E-06
Apis mellifera	140	4	0.0021	0.026
Anopheles gambiae	24	1	6.0E-4	0.036
Ciona intestinalis	104	4	0.0011	0.0027
Gallus gallus	503	33	0.0034	1.8E-08
Xenopus tropicalis	422	85	0.0026	8.6E-47
Danio rerio	643	43	0.0030	2.9E-10
Tetraodon nigroviridis	474	25	0.0028	1.4E-4
Takifugu rubripes	521	23	0.0032	0.0025
Monodelphis domestica	470	28	0.0028	2.6E-08
Bos taurus	574	40	0.0035	1.3E-06
Canis familiaris	542	44	0.0035	7.7E-09
Rattus norvegicus	583	42	0.0034	7.7E-10
Mus musculus	630	49	0.0032	2.5E-17
Macaca mulatta	550	37	0.0033	9.9E-09
Pan troglodytes	639	45	0.0036	5.7E-09
Homo sapiens	674	48	0.0033	4.8E-16

Table S1. Probabilities that U12-type introns were randomly inserted into each genome along with all parameters used to calculate those probabilities. If U12-type introns were randomly inserted a genome, one would expect the distribution of U12-type introns per gene to be binomial with parameters n = number of genes with a U12-type intron and $p = 1 - (1 - x)^{m-1}$, where x is the proportion of U12-type introns in the genome and m is the average number of introns in the genome. Organisms are grouped by phylogeny. *S. cerevisiae*, *S. pombe*, and *C. elegans* were omitted from this analysis as they lack U12-type introns. *D. melanogaster* was omitted from this analysis as there are no genes with multiple U12-type introns in that genome.

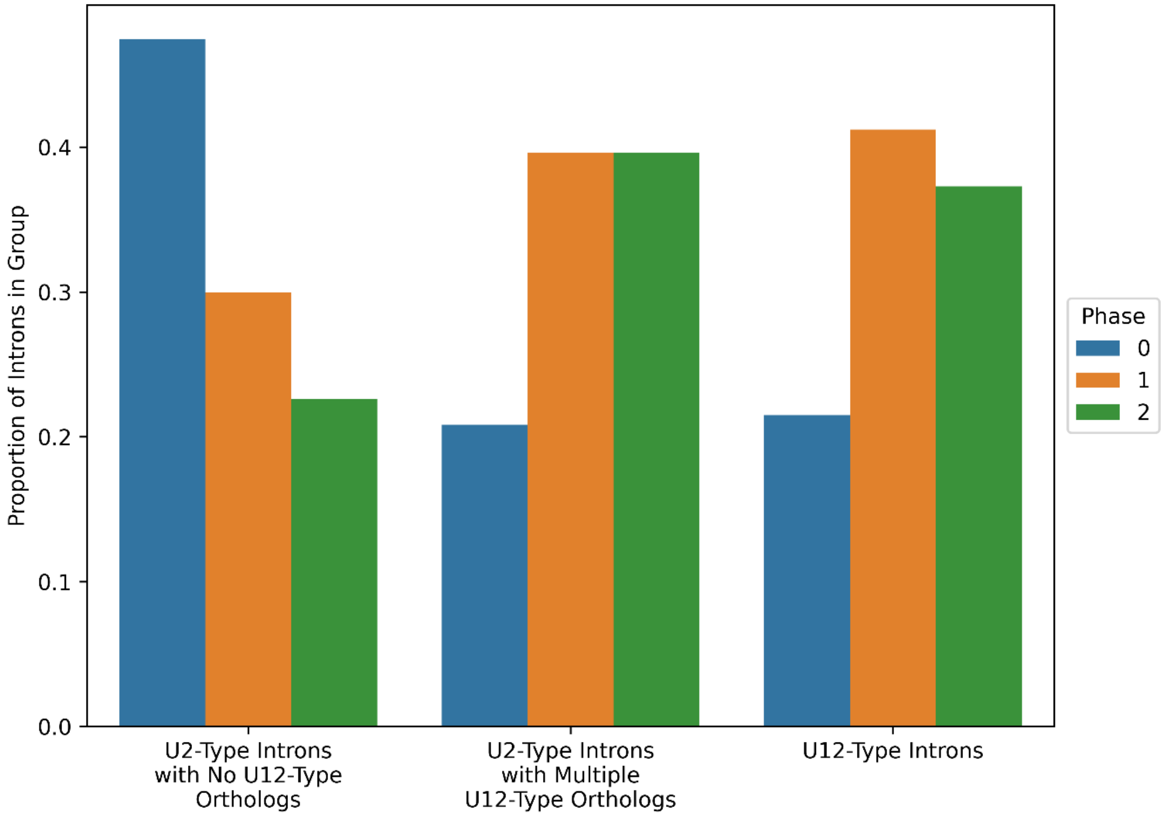

Figure S1. Distributions of intron phases (0, 1, or 2) across different sets of introns, showing similarities between putative U12-type → U2-type conversions (i.e. U2-type introns from orthologous groups containing multiple U12-type introns) and U12-type introns. (left) U2-type introns in orthologous groups where no orthologous intron is called as U12-type (n=3,348,724); (middle) U2-type introns in orthologous groups where at least two other members are called as U12-type (n=437); (right) called U12-type introns without U2-type orthologs (n=7,820).

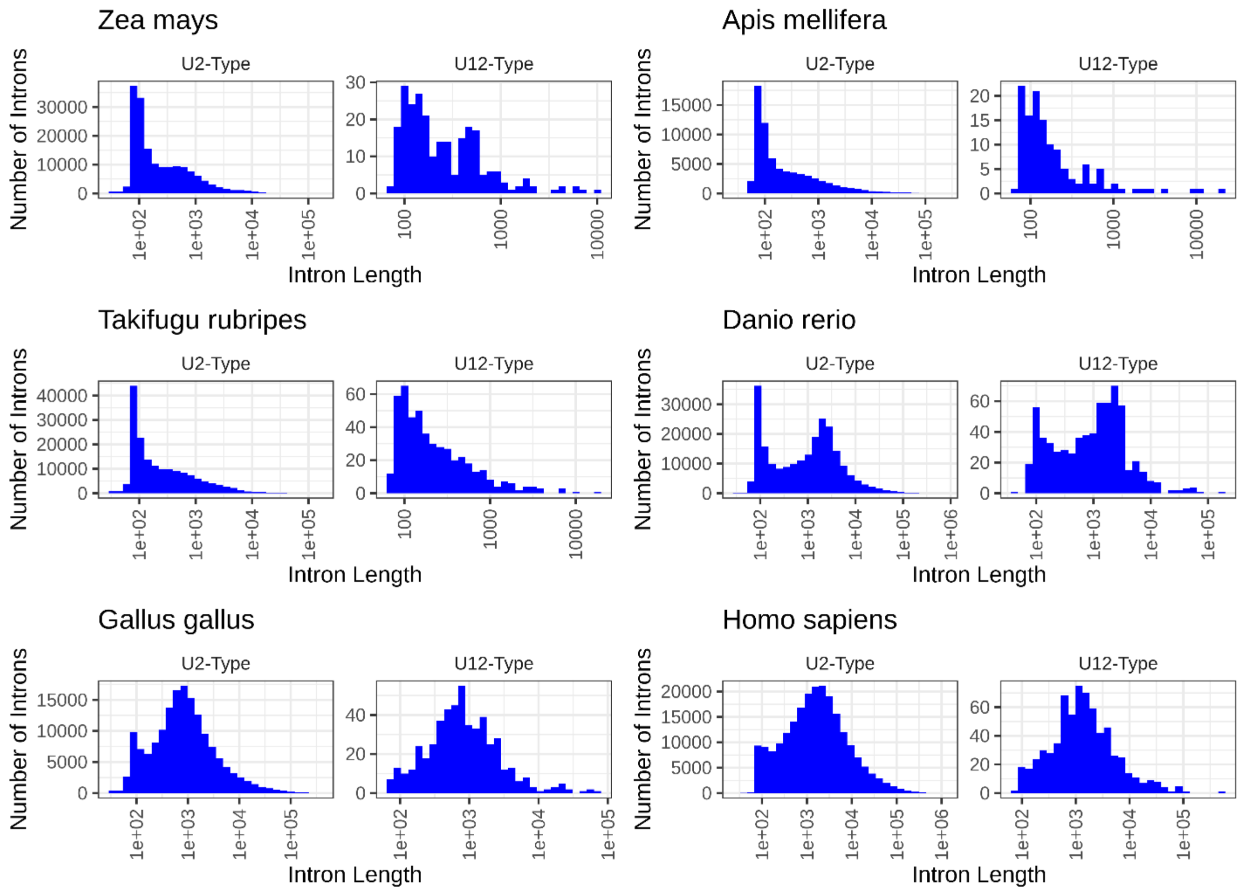

Figure S2. Distributions of intron lengths in both classes of intron in six of the genomes annotated in the IAOD. The x-axis of each plot is a log scale.

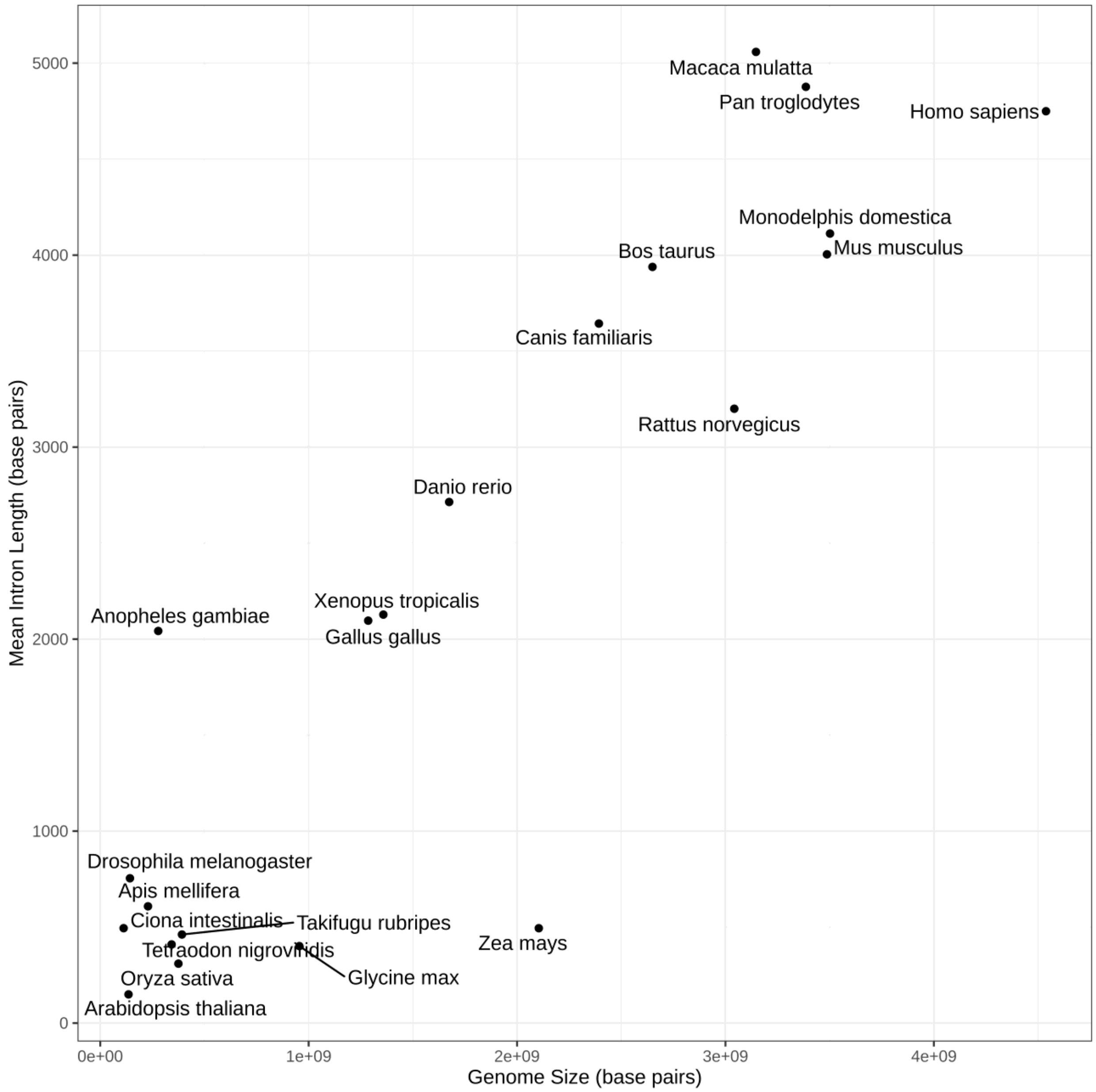

Figure S3. Relationship of genome size and mean U12-type intron length in genomes annotated in the IAOD. *Schizosaccharomyces pombe*, *Saccharomyces cerevisiae*, and *Caenorhabditis elegans* are not shown in this figure as they lack U12-type introns.

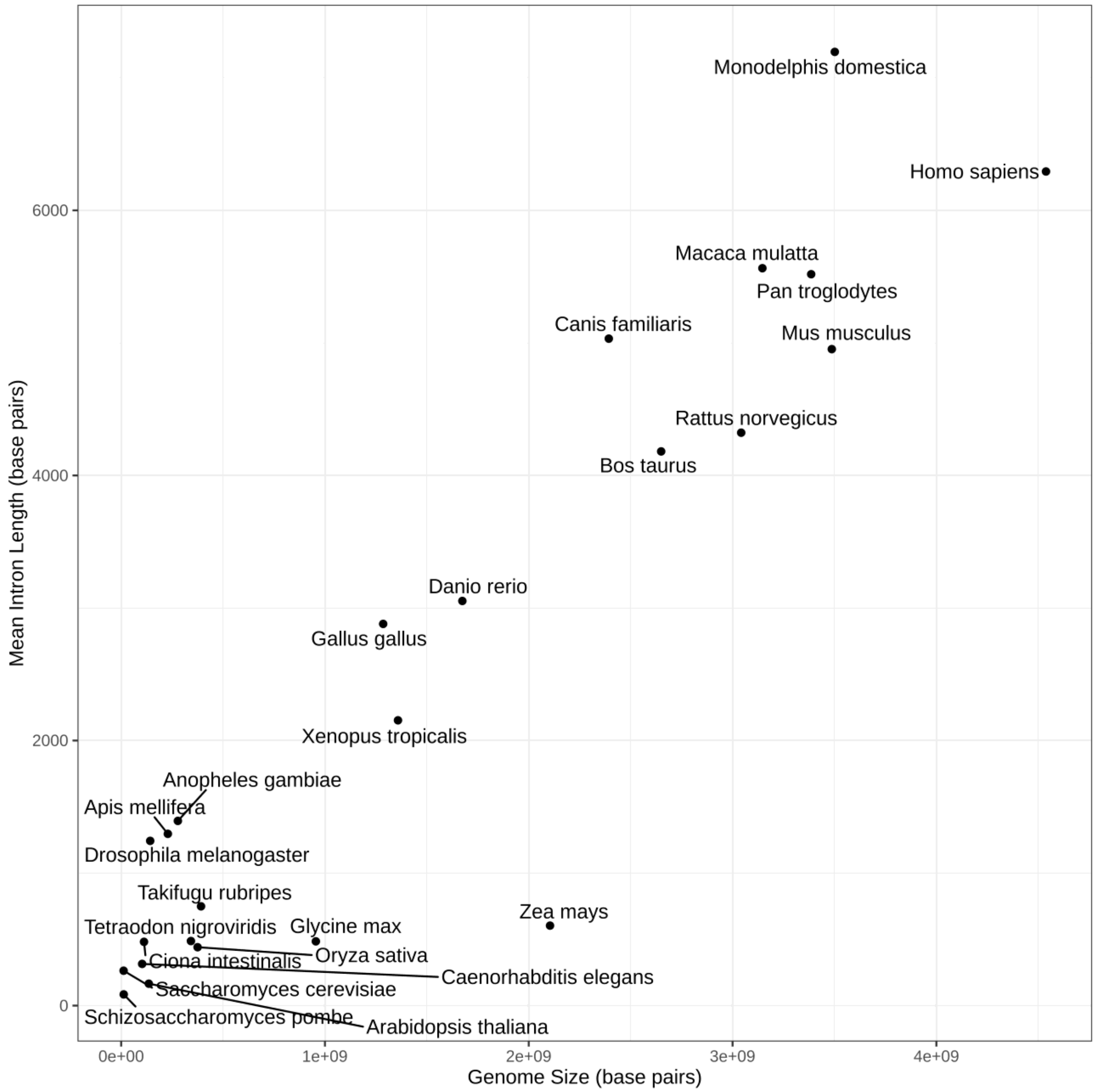

Figure S4. Relationship of genome size and mean U2-type intron length in genomes annotated in the IAOD.
